# A Defence of the Old Hospital System

**Published:** 1888-08-11

**Authors:** Sydney Waterlow

**Affiliations:** Bart., Treasurer of St. Bartholomew's Hospital.


### A Defence of the Old Hospital System.

By Sir Sydney Waterlow, Bart., Treasurer of St. Bartholomew's Hospital.

Comments have been made on what I said at a previous meeting of The Hospitals Association in reference to special hospitals. I did not say that their money was ill got and ill spent; but my experience, after fourteen years' examination of the accounts of our special hospitals, and of a comparison of them with the accounts of the general hospitals, tells me that the money collected by the special hospitals does not produce such results as the money collected by the general hospitals. The cost of the management of special hospitals is perhaps greater than that of general hospitals; but of the money spent in the special hospitals and the money spent in general hospitals, I certainly do say that the money spent in the special hospitals does not go so far in the relief of the sick poor as the money spent in general hospitals does. A very great deal might be said in reference to special hospitals. I must express my gratitude to Mr. Burdett-Coutts for the very excellent paper which he read at the April meeting of the Association. The subject is one of the greatest possible interest to those who work in hospitals, and the paper is one which everyone will thank Mr. Coutts for, and one which will be fully appreciated by all the large medical institutions of the country. I think there can be no very great divergence of opinion amongst those interested in hospitals as to the principle upon which the paper is based. If I mistake the principle Mr. Coutts will correct me; but I understood from his enunciation of the principle that it was this: that all sick persons who required medical or surgical aid ought not to receive it from charitable resources if they are in a position to pay for it. The only point in the difference of opinion is the manner in which the principle should be applied. That is much more difficult in general hospitals, especially in general hospitals based upon the constitution of a charter, or an Act of Parliament, or a will, which distinctly states that the hospital shall be directly for the relief of the sick poor, and makes no reference to the principle of payment. Mr. Burdett-Coutts has very properly paid a fair tribute to Mr. Burdett, who has had so large an experience of hospital work in every phase, and who, Mr. Coutts rightly says, was the pioneer of the Pay System. What was the first experiment? Mr. Burdett's first experiment was to establish a home hospital, where it would be self-supporting, and where persons could get relief at the smallest possible cost. I think the Pay System should be confined to one class of institution, and the absolutely charitable and voluntary relief of thousands of the sick poor should be confined to other institutions. It is where two systems are mixed together that I have found the whole objection that I could possibly raise to the system. I am not speaking without having had some experience. My experience, I may say, extends over twenty years. For eight or nine years I was chairman of the Central London Sick Asylum, where I had one hospital of 527 beds and another of 273 beds. I saw something of infirmaries, and I have been treasurer of St. Bartholomew's Hospital for 14 years, and have had opportunities of seeing, day by day, and week by week, the working of a large general hospital. Mr. Nixon has described what he has done at the London. At St. Bartholomew's, for five or six years, we have had a person who is called an inspector, and who stays in the receiving-room and sees everyone who comes in. If any gentleman present would come some morning at nine o'clock and watch the persons come in, you would not have any doubt of nine out of ten of them, as to their inability to pay. But if you come to the proof, you would find that the tenth was a case which ought not to be charitably relieved. We say to that person, "You can have relief to-day," and then the inspector makes it his duty to go to the address which has been given and to ascertain the kind of home in which the patient lives, and probably sees someone connected with him or her, if he does not see the patient, and if they cannot afford to pay, they are too much ashamed to come any more. In that way, I believe we keep out almost every one who is undeserving to receive the great charity that is given in the hospital. I am quite willing to admit that there may be some mistake in this examination, and that some people do obtain relief who ought not to, but it is a very small proportion, and it is better that we should relieve one undeserving person than turn away four or five deserving persons. A great deal has been said in reference to self-help, and I was much pleased by hearing the observation that you must not push self-help too far. There is scarcely a man in London who has striven more than I have to make the working people understand that the first necessities of life they must provide for themselves. That is a principle which I have most constantly urged; but it must be remembered that medical and surgical sicknesses and accidents constantly and generally arise without the slightest warning and without the people who suffer by them having the slightest warning, or being able to make the slightest possible preparation. They are constantly happening, and if they were not relieved at the time, the bread winner would be very frequently pauperised, and a fresh burden cast upon the community—both of which disasters are prevented by the immediate treatment of the disease or accident when it enters the door of the hospital. I quite agree with a gentleman who said that the managers of large hospitals are responsible for seeing that the charities entrusted to them are properly administered, and that they are not used for the relief of those who are undeserving; and I should blame myself very much indeed if in the management of our large hospitals we did not take every possible precaution. But I rather object, and I think it is better to say so, to the conversion of hospitals. They were built by those who left the money entirely for the sick poor. Now I should be told that the only alternative, when a great hospital like Guy's drifts into pecuniary difficulties, is either to fill up the beds with patients who can pay, or to let them remain empty. I think the governors of Guy's Hospital, and the governors of all hospitals which were entirely free and have become ordinary paying hospitals should remember that every effort should be made to restore these hospitals to their original condition. They were a gift to the poor, and not a gift to those who could afford to pay. I believe that if those who want money for really good charitable purposes can show that the money, if bestowed, will be well managed and utilized to the best advantage, no large institution having such objects as the relief of the sick poor need appeal to the British public in vain. I agree with the course which the London Hospital has adopted in confining itself entirely to free patients and throwing itself upon the public. They have adopted that course and have been successful, and they keep their beds as full as any large hospital. I cannot, in conclusion, say that I am in favour of the Pay System, but I do not think, from my experience—and I have had opportunities of seeing the affairs of every hospital in London—that it will be found entirely satisfactory or successful unless you keep the pay patients in one part of the hospital and the free patients in another.

